# Rocky Mountain Spotted Fever Associated With Cardiac Arrhythmias

**DOI:** 10.7759/cureus.42288

**Published:** 2023-07-22

**Authors:** Zeib Syed, Christian Scott, Bao Nguyen, Ei Phyu

**Affiliations:** 1 Internal Medicine, LewisGale Medical Center, Roanoke, USA; 2 Internal Medicine, LewisGale Medical Center, Salem, USA

**Keywords:** rickettsia, tick-borne infections, life threatening arrhythmia, heart block, rocky mountain spotted fever

## Abstract

Rickettsial infection, known as Rocky Mountain spotted fever, is a challenging diagnosis as early clinical manifestations are difficult to distinguish from viral illnesses. Symptoms at presentation depend on the organs involved, ranging from a skin rash as evidence of vascular damage to prerenal azotemia, respiratory failure, hepatic injury, or encephalitis. We report an unusual case of an otherwise healthy 83-year-old female whose serologies tested positive for *Rickettsia rickettsii*, which led to cardiac dysrhythmia, i.e., the new onset of atrial fibrillation associated with conversion pauses. The patient was treated with antibiotics and ultimately underwent permanent pacemaker placement with resolution. This case highlights cardiac dysrhythmia as a late and severe manifestation in patients diagnosed with rickettsial illness.

## Introduction

*Rickettsia rickettsii*, the cause of Rocky Mountain spotted fever (RMSF), is a common and curable tick-borne illness found primarily in the United States. The American dog tick, Dermacentor variabilis, is responsible for the spread of this infection in the eastern United States, while the Rocky Mountain wood tick, *Dermacentor andersoni*, spreads this infection in the western United States. Rickettsia organisms are typically released into the dermis with salivary excretions after six or more hours of feeding, leading to lymphohematogenous dissemination. Clinical manifestations are typically nonspecific and similar to nonspecific viral illnesses: fever, myalgias, nausea, vomiting, and headaches. As the course of the disease progresses, clinical manifestations include the characteristic petechial rash that begins peripherally at the wrist and ankles and spreads toward the chest. *Rickettsia rickettsii* is known to affect endothelial cells specifically and therefore can affect multiple organ systems, including neurologic, respiratory, cardiac, digestive, and renal. [1] With respect to cardiac infiltration, this case presents a previously healthy 83-year-old female presenting with an RMSF infection that developed into new-onset atrial fibrillation with conversion pauses followed by a stroke.

## Case presentation

**Figure 1:**
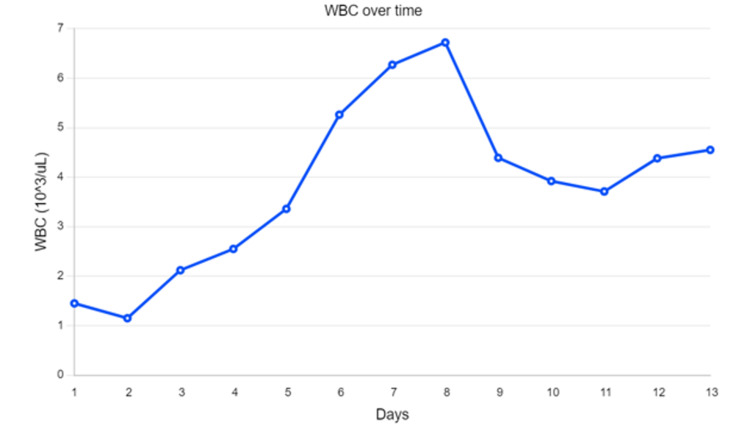
Leukocyte count over the course of hospitalization The patient initially presented with leukopenia which resolved over time with the addition of antibiotics (doxycycline and cephalosporin) which were started between days 2 and 3. Note that her leukopenia resolved between days 5 and 6.

**Figure 2:**
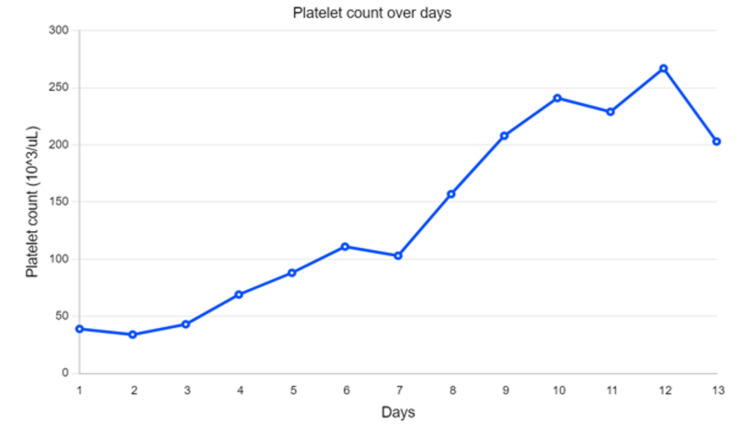
Platelet count over the course of hospitalization The patient initially presented with thrombocytopenia which resolved over time with the addition of antibiotics (doxycycline and cephalosporin) which were started between days 2 and 3. Note that her thrombocytopenia resolved between days 7 and 8.

An 83-year-old woman with past medical history of arthritis presented to the emergency room with symptoms of decreased appetite, fatigue, dry cough, and body aches ongoing for three days. When she presented to the hospital, she was noted to have leukopenia with bandemia and thrombocytopenia as well as a measured fever of 104 °F (Table 1). The imaging of the chest was unremarkable. Urinalysis did not show any infectious process but did reveal dark concentrated urine reflecting her poor appetite during this time. Labs were also notable for transaminitis and the subsequent sonogram of her right upper abdomen showed some degree of hepatic parenchymal disease and trace ascites (Table 2). The patient subsequently had *R. rickettsii*, Lyme disease, RMSF, and ehrlichiosis panels ordered; *R. rickettsii *IgM returned positive. Of note, the patient also had a parvovirus IgG which was also positive. Labs for Lyme disease and ehrlichiosis were ordered to rule out tickborne illnesses, and both were negative. The patient was started on cefepime and doxycycline and Infectious Disease was consulted whereupon the patient was switched to cefoxitin and doxycycline for the remainder of her clinical course. As illustrated in Figures 1-2 below, the patient did have a resolution of her white count and platelet count with the progression of treatment. The patient did not have development of rash during her hospital course.

**Table 1:** Complete blood count with differential The patient was started on doxycycline between days 2 and 3 as noted by the asterisks above. The elevated D-Dimer on day 3 likely reflects acute phase reactant in the setting of acute infection. There is an appreciable recovery in the patient's bandemia as well as her leukopenia and thrombocytopenia.

Complete blood count	Day 1	Day2*	Day 3*	Day 4	Day 5	Day 6	Day 7	Day 8	Day 9	Day 10	Day 11	Day 12	Day 13
White blood count	1.45	1.15	2.12	2.55	3.36	5.26	6.27	6.72	4.39	3.92	3.71	4.38	4.55
Red blood count	4.33	3.89	4.03	3.71	3.88	4.37	4.38	4.18	4.29	3.86	3.79	3.69	3.79
Hemoglobin	12.2	11.0	11.4	10.6	11.2	12.2	12.4	11.8	12.0	11.1	10.6	10.4	10.6
Hematocrit	36.6	33.9	35.2	33.1	34.3	38.6	39.1	36.3	37.3	33.6	33	31.9	32.9
Mean corpuscular volume	84.5	87.1	87.3	89.2	88.4	88.3	89.3	86.8	86.9	87.0	87.1	86.4	
Platelet	39	34	43	69	88	111	103	167	208	241	229	267	203
Neutrophil %	68	64	71	70	60	61	36	54	60	65	65	54	60
Band neutrophils %	14	26	19	10	15	1		1					
Lymphocytes %	6		5	6	16	24	41	36	25	16	13	23	20
Atypical lymphocytes %	7	4	1	2	1	4	6	3	3				

**Table 2:** Comprehensive metabolic panel The patient's electrolyte abnormalities as well as her transaminitis had resolved with the progression of her clinical course and treatment of her infection. Her phosphorous was noted to be persistently low and suspected to be a component of refeeding syndrome as well.

Comprehensive metabolic panel	Day 1	Day 2	Day 3	Day 4	Day 5	Day 6	Day 7*	Day 8	DAY 9	Day 10	Day 11	Day 12	Day 13*
Sodium	133	136	141	144	143	145	142	138	134	138	135	137	136
Potassium	3.5	3.5	4.0	4.5	4.0	3.6	4.2	3.5	4.0	4.2	4.4	4.1	4.1
Chloride	95	99	110	114	110	111	109	103	101	105	104	104	29
Bicarbonate	27	27	24	23	25	28	26	29	28	29	29	28	13
Blood urea nitrogen	24	19	23	28	28	17	13	12	19	12	13	12	13
Creatinine	0.99	0.72	0.65	0.77	0.91	0.59	0.61	0.38	.96	0.49	0.48	0.49	0.52
Glucose	110	96	91	93	134	109	93	99	178	131	146	108	135
Calcium	8.6	8.2	7.7	7.3	7.5	8.0	7.7	7.5	7.4	8.0	8.0	8.0	7.9
Phosphorous			3.0	2.7	1.4	1.6			1.7	2.4	3.4		
Magnesium			2.4		2.4			2.0	2.4	2.2	1.9		
Total bilirubin	0.7	0.6	0.5	0.5	0.4	0.6			0.5		0.3		
Direct bilirubin			< 0.1			0.20							
Aspartate aminotransferase	159	201	327	238	186	138			45		39		
Alanine aminotransferase	91	86	99	78	50	43			29		26		
Alkaline phosphatase	352	289	262	253	227	213			185		173		
Troponin			247-> 238 -> 144						26				
Total protein	6	5.1	4.9	4.1	4.5	5			5.2		5.3		
Albumin	3	2.6	2.8	1.7	1.7	1.8			1.8		1.8		
Globulin	3	2.5		2.4	2.8				3.4		3.5		
Lipase	106												
Thyroid-stimulating hormone	1.310												

**Figure 3:**
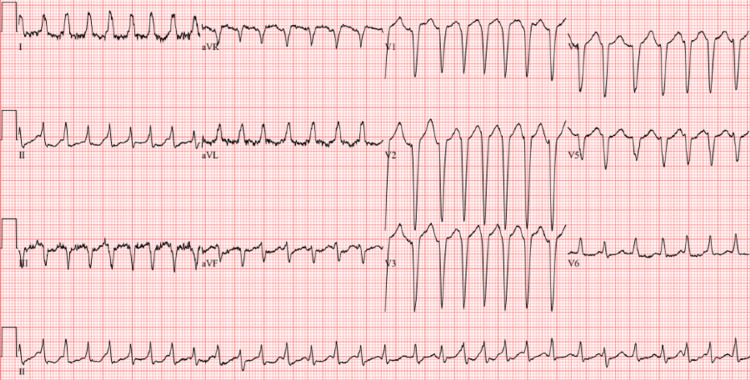
Electrocardiogram of the patient demonstrating a wide complex, irregularly irregular rhythm

On day 2 of hospitalization, the patient developed atrial fibrillation with rapid ventricular response with a heart rate of 190 to 200 and her systolic blood pressure dropped to 80 (Figure 3). She was subsequently sent to the ICU where her blood pressure and heart rate were closely monitored.

On day 7, renally-dosed eliquis was initiated on the patient. She was noted to be stable with the use of metoprolol titrated up for rate control and stepped down thereafter. Throughout this time, she was continued on doxycycline and her leukopenia and thrombocytopenia were noted to slowly resolve (as seen in Table 2).

**Figure 4:**
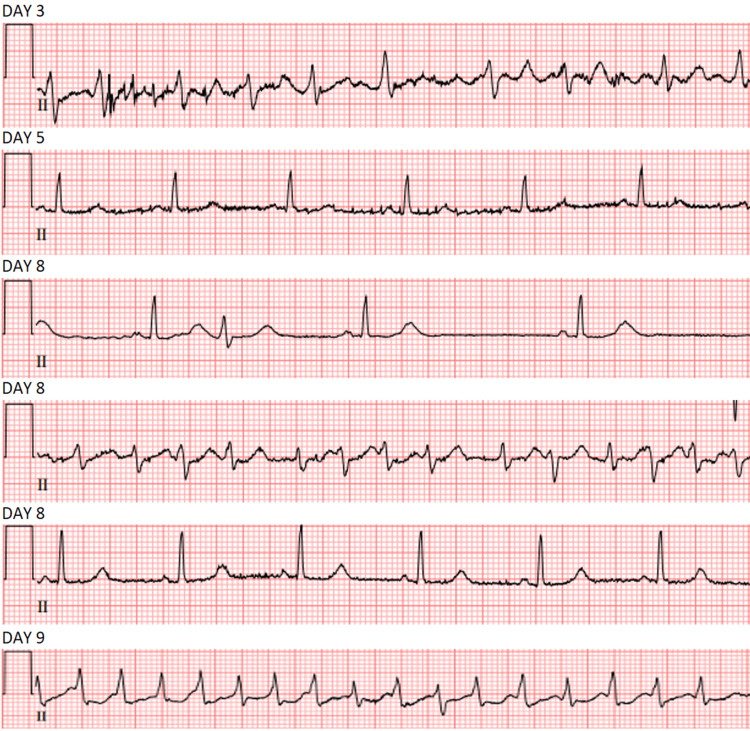
The patient's electrocardiograms throughout the clinical course with a particular focus on lead II The patient initially had new-onset atrial fibrillation and it is noted that she had been going from atrial fibrillation with rapid ventricular response to sinus rhythm after her conversion pauses. Conversion pauses were noted to be 5.5 seconds at their longest, however, it is not pictured here.

**Figure 5:**
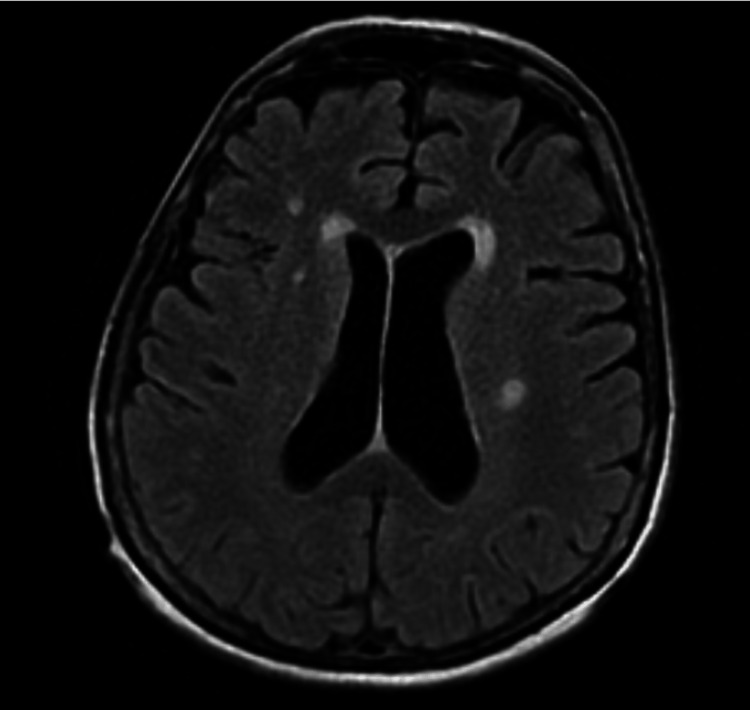
MRI brain of the patient demonstrating an acute stroke to her corona radiata in the setting of her irregular rhythm with conversion pauses and resumption of sinus rhythm

On day 9 of hospitalization, the patient was noted to have an irregular wide complex tachycardia and subsequently developed pauses increasing in frequency and duration with the longest noted to measure at 5.5 seconds. Cardiology remarked that the patient had atrial fibrillation with rapid ventricular response with concurrent conversion pauses back to sinus rhythm. The patient was treated with oral amiodarone in addition to metoprolol and eventually was transferred to the ICU and started on an amiodarone drip. The patient was noted to be fairly symptomatic throughout these events. On day 10, she had a 24-beat run of ventricular tachycardia and soon after had a stroke alert called due to right-sided hemiparesis and worsening mentation (Figure 4). She had an MRI done of the brain which revealed acute non-hemorrhagic infarct involving the left corona radiata (Figure 5). Electrophysiology was consulted and given the patient's frailty and clinical picture, the risks outweighed the benefits in terms of intervention. The patient's heart rate was noted to be controlled on the current regimen and she was discharged to follow-up with cardiology. In the outpatient setting, she was noted as having sinus bradycardia on an EKG and eventually stopped taking her rate and rhythm control medications, and has had no cardiac-related issues since.

## Discussion

*Rickettsia rickettsii* is a gram-negative coccobacilli, an obligate intracellular organism and the causative agent for RMSF, which has been described in the literature as the most lethal form of rickettsiosis [2]. Rocky Mountain spotted fever was initially recognized in the early 1800s and was noted to be prevalent in the Bitterroot Valley area of Montana. Today, cases have steadily risen in the past few decades with incidence reaching around 5000 as of 2019 and over 50% of cases happening in Arkansas, Missouri, North Carolina, Tennessee, and Virginia [3]. *Rickettsia *infections usually impact the vascular endothelium of vessels, causing inflammatory responses and subsequent systemic disease. More commonly, there have been reports of fever, muscle pains, confusion, rash, thrombocytopenia, leukopenia, and cardiovascular symptoms [4,5]. With regards to cardiovascular symptoms, there have been cases throughout the years commenting on Rickettsia and tick-borne illnesses as causes of cardiovascular arrhythmias. Since the late 1980s, when the first human case of tick-borne illness was described, *Ehrlichia* has been one of the more commonly noted cases. A 46-year-old male from Arkansas was exposed to multiple tick bites and had subsequent atrial flutter in his clinical course [6]. The literature supports that the pathophysiology responsible for the clinical course of *Ehrlichia*, not unlike that of *Rickettsia*, seems to be inflammatory in nature [4]. Apart from Ehrlichia, there have been cases of different *Rickettsia* species being responsible for arrhythmias. In the case of a 58-year-old man affected by *Rickettsia conorii* leading to a Mediterranean spotted fever, the patient developed a maculopapular rash and black eschar, and subsequent atrial fibrillation. This case supports the recommendation that heart function be monitored in patients afflicted by this disease, even in the absence of underlying risk factors [7]. Rocky Mountain spotted fever, as seen in our patient, has been shown to rarely impact the myocardium and mimic symptoms of acute coronary syndromes. In our case, we had an elderly female presenting with leukopenia, thrombocytopenia, fatigue, and muscle pains with positive serologies for* R. rickettsii* leading to RMSF, but she interestingly did not develop a rash throughout her clinical course. Later on, the patient developed new-onset atrial fibrillation and eventually had conversion pauses. As noted previously, there have been some cardiovascular manifestations as a result of RMSF inflammatory modulation. The relationship between* R. rickettsii *and atrial fibrillation with cardiac arrhythmias has not yet been demonstrated in the literature. Our patient eventually responded to an antibiotic course with correction of labs and was treated with amiodarone, metoprolol, doxycycline, and cephalosporin with eventual resolution.

## Conclusions

This is a case of an unfortunate woman exposed to RMSF with the development of subsequent new-onset arrhythmias which seemed to respond to treating the underlying infection. The relationship between tick-borne illnesses and cardiac arrhythmias underscores the significance of early intervention and the critical role telemetry can play in this process. By advocating for the use of telemetry in suspected tick-borne infections, providers can act early to increase the chances of positive outcomes, reduce the severity of potential cardiac complications, and improve the overall management of the disease. In addition, this case also notes the importance of early anticoagulation to mitigate stroke and the importance of monitoring the recovery of platelet count as treatment progresses.
